# Using ICPC-2 Standard to Identify Thai Zingiberaceae of Pharmacological Interest

**DOI:** 10.3390/plants9070906

**Published:** 2020-07-17

**Authors:** Methee Phumthum, Henrik Balslev

**Affiliations:** 1 Department of Pharmaceutical Botany, Faculty of Pharmacy, Mahidol University, Bangkok 10400, Thailand; 2 Sireeruckhacharti Nature Learning Park, Mahidol University, Nakhon Pathom 73170, Thailand; 3 Department of Biology, Faculty of Natural Science, Aarhus University, 8000 Aarhus, Denmark; henrik.balslev@bios.au.dk

**Keywords:** *Zingiber*, *Curcuma*, digestive, medicinal, ethnobotany, gingers, carminative, gastritis, International Classification of Primary Care-2, ICPC-2

## Abstract

The Economic Botany Data Collection Standard (EBDCS) is a widely used standard among ethnobotanists. However, this standard classifies ethnomedicinal uses into categories based on local peoples’ perception. It is difficult to apply in pharmacological research. The International Classification of Primary Care (ICPC), now updated to ICPC-2, is more related to medical terms, but is rarely used among ethnobotanists. This study aims to apply the ICPC-2 to classify metadata of the ethnomedicinal uses of Zingiberaceae plants in Thailand, in order to identify important medicinal taxa for future research. Data on the ethnomedicinal uses of Thai gingers were collected from 62 theses, journal articles, scientific reports and a book, published between 1990 and 2019. Scientific plant names were updated using The World Checklist of Vascular Plants (WCVP) website. Informant Consensus Factor (ICF) was used to identify the medicinal issues commonly treated with gingers, and the Cultural Importance Index (CI) was used to identify species that might have pharmacological potential. We found records of 76 ginger species with ethnomedicinal uses, and together they had 771 use reports. The gingers were commonly used for treatments related to digestive system conditions, particularly abdominal pain and flatulence. Gingers remain exceedingly important in Thai ethnomedicine, with a high number of useful species. They are used to treat a variety of health conditions, but most commonly such ones that are related to the digestive system. Apart from the popular studied ginger, *Curcuma longa*, we identified a number of other useful gingers in Thailand.

## 1. Introduction

Traditional ethnomedicinal knowledge is not only important to local people who own the knowledge, but is also important for people around the world, as sources of knowledge for modern drug development [1]. So, ethnobotanical study plays an important role in supporting findings. Using similar plants for the same or similar purposes as those of people living in different areas implies the efficacy of the plants [2]. For these reasons, being guided by ethnomedicinal uses would facilitate more opportunities for drug developments. Connecting the ethnomedicinal study and the modern medical study needs an appropriate link. Most of the ethnobotanical studies use the *Economic Botany Data Collection Standard* (EBDCS) [3], which classifies uses into categories based on local peoples’ perceptions [4]. Hence, the EBDCS categories would not be appropriate guides for drug development. The *International Classification of Primary Care* (ICPC), which is now updated to ICPC-2 [5], is an interesting standard supported by the World Health Organization (WHO). The standard provides categories more related to modern medical terms. This study, therefore, proposes to use this standard in classifying ethnomedicinal use reports, so as to identify Zingiberaceae plants as candidates for drug development research based on ethnomedicinal knowledge in Thailand.

Zingiberaceae (the ginger family) comprises approximately 50 genera and 1600 species of aromatic, perennial, rhizomatous herbs [6]. Of these, 1300 species in 45 genera are found in Asia, and much fewer species are found in other tropical regions, such as tropical Africa (90 species, 4 genera) and tropical America (55 species, 1 genus) [7]. Species of Zingiberaceae—from now called “gingers” in this paper—produce food, spices, dying materials, perfumes and cosmetics [8,9,10]. Importantly, many gingers have been used in ethnomedicine, and many have therapeutic properties [11]. Moreover, some gingers have compounds that have been used to produce novel medicines, such as curcumin from *Curcuma longa* L. [12], gingerol from *Zingiber officinale* Roscoe [13], and various compounds from species of *Alpinia* [14].

Thailand is part of the Indo-Burmese biodiversity hotspot [15], and houses over 11,000 plant species [16], including over half of the world’s ginger genera [7]. Many gingers have had an intricate relationship with Thai culture since time immemorial. Several Thai food recipes use gingers as spices, such as the well-known Tom-Yam, which is cooked using rhizomes of galangal *Alpinia galanga* (L.) Wild. Other species, such as finger roots (*Boesenbergia rotunda* (L.) Mansf.), ginger (*Zingiber officinale* Roscoe) and turmeric (*Curcuma longa* L.) are important components in different Thai curry pastes. Some gingers, especially species of *Globba*, are used in rituals because of their pleasing scents, and many of them have propitious local names. Although gingers have many different uses, their medicinal properties may be the most important of all. A study of plant families in Thailand showed that species of the ginger family are among the most ethnobotanically important in the country [17]. Many studies of plant uses have appeared since ethnobotany was introduced as a research field into the Thai academic system in 1990. Nevertheless, those studies mostly focused on uses other than the medicinal uses of gingers, and there were only a few use reports of medicinal gingers in each study. A few publications, however, deal exclusively with the use of gingers as medicine. For instance, there is a Master’s thesis on traditional knowledge of the cultivation and uses of gingers, but it is limited to *Zingiber officinale* [18]. Another study of the medicinal and auspicious plants in the ginger family focused on chemotaxonomy, but the study was limited to one subdistrict in Chiang Mai province [19]. One study did cover the ethnomedicinal uses of gingers in many parts of Thailand, and documented 77 use reports from 58 species [20]. Here, we mined all available ethnomedicinal references for uses of gingers in Thailand, and we identify those species that are potential candidates for further pharmacological study using the ICPC-2 standard. Specifically, we aim to answer the following questions: (1) Which species and genera of gingers have been used as medicine in Thailand? (2) Which medicinal categories were most commonly treated by gingers? (3) Which ginger species have the potential for specific treatments, and could be candidates for further pharmacological research?

## 2. Results

### 2.1. Genera, Species, and Their Numbers of Use Reports

The data search generated 771 ethnomedicinal use reports for the ginger family in Thailand. These use reports were associated with 76 species in 13 genera. Mostly, the plant part used was the rhizome. *Zingiber montanum* (J.Koenig) Link ex A.Dietr. had the highest number of use reports (108), followed by *Curcuma longa* L. (62), *Zingiber officinale* Roscoe (53), *Alpinia galanga* (L.) Willd. (43), *Kaempferia parviflora* Wall. ex Baker (43) and *Zingiber ottensii* Valeton (41). The next group of species, *Kaempferia galanga* L., *Zingiber zerumbet* (L.) Roscoe ex Sm., *Curcuma zedoaria* (Christm.) Roscoe, *Kaempferia rotunda* L., *Curcuma aeruginosa* Roxb., *Alpinia malaccensis* (Burm.f.) Roscoe and *Boesenbergia rotunda* (L.) Mansf., had 20–38 use reports, while the remaining 65 species had 1–15 use reports (Table S1).

Among the genera, *Zingiber* had the highest number of use reports from 12 species. The genus *Curcuma* had the highest number of medicinal species in the family, but with fewer use reports than *Zingiber*. Seven *Kaempferia* species provided 117 use reports, and there were 104 use reports from 10 *Alpinia* species, while the other genera had fewer than 30 use reports (Table S1).

### 2.2. Use Categories and Uses

**Figure 1:**
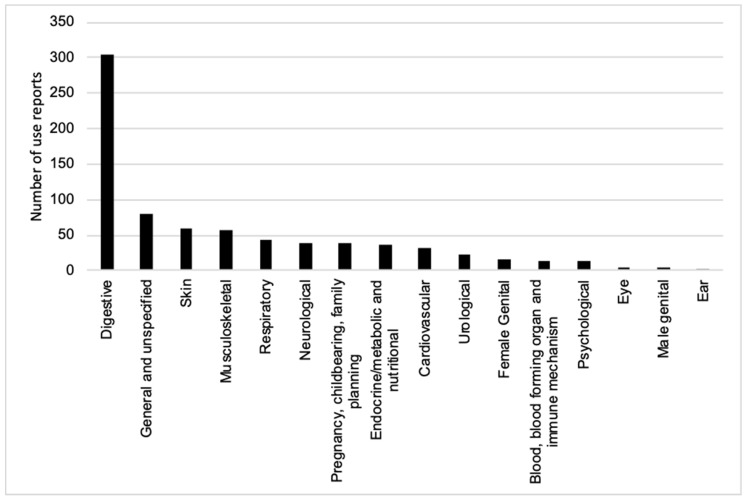
The numbers of use reports for Thai ethnomedicinal gingers in each medicinal use category following the *International Classification of Primary Care* (ICPC-2).

Approximately 40% of the 771 use reports pertained to treating disorders in the digestive system. The category of general, unspecified health problems had a much lower number of use reports, followed by skin-related symptoms, musculoskeletal system disorders, respiratory system disorders and neurological disorders. The categories of pregnancy, childbearing and family planning, endocrine/metabolism and nutrition, cardiovascular issues, urological issues, female genital issues, psychological issues, and blood, blood forming organ and immune mechanism, together had only a few use reports (Table S1 and Figure 1).

### 2.3. Informant Consensus Factors (ICF)

Among the ICPC-2 categories, digestion-related conditions had the highest ICF score. Clusters of health conditions related to general and unspecified, skin system disorders, the category of pregnancy, childbearing and family planning, and that of male genital disorders also had high ICF values. The next groups with slightly lower values were musculoskeletal conditions, and disorders related to the ear and eye. The remaining categories had ICF values below 0.5 (Table 1).

### 2.4. Gingers Used to Treat Digestive System Disorders

There were 306 use reports related to treating 11 symptoms in the ICPC-2 category of digestive disorders (Table S1), of which more than one-third were used to treat flatulence, while 27% mentioned the treatment of abdominal pain. Of the reports, 9% mentioned that gingers were used to treat gastritis, which is a condition related to abdominal pain; however, the original reports specifically mention uses of ginger for the treatment of gastritis. Uses of gingers as a laxative, and to treat diarrhea and colitis, made up approximately 7% of all use reports, while issues of indigestion, food poisoning, antiemetic, bile tonic and hepatopathy each had very a few use reports (Table S1).

We found very high ICF values for treatments of flatulence and abdominal pain. The ICF values for treating diarrhea, peptic ulcers, constipation and colitis were also high, and varied between 0.40 and 0.50 (Table 2).

### 2.5. Cultural Importance Index (CI) of Gingers Used to Treat Digestive Health Conditions

Some ginger genera had noticeably high CI values for specific treatments. Species of *Curcuma* and *Zingiber* had high CI for the treatment of flatulence (nearly 11%). Our “pseudoinformants” also agreed that the genus *Curcuma* was useful for the treatment of abdominal pain and peptic ulcers. *Kaempferia* species were used to treat flatulence, abdominal pain, diarrhea and peptic ulcer (Table 3).

The CI values for *Zingiber montanum* (J.Koenig) Link ex A.Dietr. and *Curcuma longa* L. ranked among the top five for treatments of abdominal pain, colitis, diarrhea, flatulence, constipation and peptic ulcers. Different gingers were used for different treatments. *Zingiber montanum* had the highest CI value for flatulence. For the treatment of peptic ulcers, the highest FL was for *C. longa*, while *Z. montanum* and *Z. ottensii* ranked as the top species for the treatment of abdominal pain (Table S2).

## 3. Discussion

Although this meta-analysis is based on data obtained from a limited number of references, our information certainly covers what is available on the subject, as we screened theses and reports from all Thai higher educational institutes. Moreover, the references also include publications in journal articles, especially from Thai journals, of which many were published in the Thai language.

For the number of ethnomedicinal species and the number of use reports, we found that these 62 references documented 76 ginger species, that together had 771 use reports. As the treatment of the ginger family for the Flora of Thailand remains to be finished, we compared species in our list to the listing found in the book *Gingers of Thailand* [7]. Plant names in that book were mostly the same as the plant names on the WCVP website [21], and only 13 names from the book needed to be updated (2 *Alpinia* spp., 2 *Amomum* spp., 5 *Globba* spp., *Hedyhium* sp., *Hornstedtia* sp., *Kaempferia* sp. and 2 *Zingiber* spp.). It is interesting that the data gathered for this study include many species that were not listed in the book (1 *Alpinia* sp., 2 *Amomum* spp., 1 *Boesenbergia* sp., 4 *Curcuma* spp., 1 *Etlingera* sp., 1 *Gagnepainia* sp., 1 *Globba* sp., 1 *Rhyncanthus* sp., 1 *Stahlianthus* sp. and 1 *Zingiber* sp.) [7] (Tables S1 and S2). This suggests that some knowledge has been exchanged between areas, or adopted from other countries. Some gingers in our list, which are not listed in *Gingers of Thailand,* were also used as medicine in China (Hong et al., 2015) [22]. Further, some species in our list may not have been taxonomically recorded in Thailand at the time the book was published in 2006. Likewise, the synopsis of the genus *Zingiber* in Thailand, published in 1999 and based on herbarium specimens, includes fewer *Zingiber* spp. than the book does [7,23].

The 771 use reports from 76 ginger species is a much higher number than in the previous ethnomedicinal studies of Thai gingers, which mentioned only 77 use reports from 58 species [20]. Still, the total numbers of medicinal gingers used in Thailand could be even higher, as suggested in our study of the entire Thai ethnomedicinal flora [24], not least because each village in the country has developed its own unique knowledge [25]. Moreover, we found references that record useful species without mentioning specific uses, while some publications mentioned only Thai names of the gingers. Therefore, more work in more villages in different regions is needed to fill this gap.

Gingers have different morphological characteristics, and each species produces a unique scent. This is a feature that helps to recognize and identify ginger species. As most of the traditional knowledge is passed on orally from one generation to the next, and the young generation learn from older generations, the less experienced can better remember species that are easy to identify. This would help villagers to recognize a number of uses of gingers. Moreover, easy accessibility is one of the factors that makes people select plants for use [26]. Many gingers are widely known in Thailand. *Curcuma longa* L., *Zingiber officinale* Roscoe, *Kaempferia parviflora* Wall. ex Baker and *Kaempferia galanga* L. can be found in all local fresh markets, and nowadays they can also be found on the shelves of many supermarkets. Gingers are spices in all household kitchens. Therefore, not only the local healers but also other people know them and can identify them. However, it is interesting that *Zingiber montanum* (J. Koenig) Link ex A. Dietr., which is not a main ingredient or spice in local cuisines, had the highest number of use reports.

The ICPC-2 standard is a good standard of the classified ethnomedicinal uses of gingers in Thailand. We found that many reports mentioned that gingers were used for the treatment of digestive system disorders (Figure 1). For this reason, we suggest ethnobotanists use this standard for future classification. However, based on this study, the standard still has some limitations with regards to classifying some reports from metadata; for example, the symptom of abdominal pain, from local peoples’ reports, is complicated. It is difficult to specify which organ is affected. The symptom covers all internal organs (digestive tract) in the abdominal cavity. However, abdominal pain mostly refers to stomach ache or gastritis (many references in the Supplementary Materials). In such a case, we suggest it is important to collect more clear information in the field. From our results, the plants that were commonly used for treatments of abdominal pain were *Zingiber montanum* (J.Koenig) Link ex A.Dietr., *Kaempferia galanga* L., *Curcuma longa* L., *Alpinia galanga* (L.) Willd. and *Zingiber ottensii* Valeton, whereas the species used for treatments of flatulence were *Zingiber montanum* (J.Koenig) Link ex A.Dietr., *Zingiber zerumbet* (L.) Roscoe ex Sm., *Zingiber ottensii* Valeton, *Curcuma longa* L., *Boesenbergia rotunda* (L.) Mansf., *Kaempferia parviflora* Wall. ex Baker, *Curcuma aeruginosa* Roxb., *Stahlianthus campanulatus* Kuntze and *Zingiber officinale* Roscoe (Table S2). These species were used not only in Thailand, but also for the same purpose in other countries, such as Cambodia [27], India [28], Papua New Guinea [29], Indonesia [30] and the Philippines [31].

Ginger species have a long history in academic research. There are thousands of results from a search in the Scopus database using the keywords ‘Zingiberaceae’ and ‘medicinal plants.’ However, most research on the pharmacological uses of ginger species focuses on *Curcuma longa* L. Our analysis suggests that many gingers may have the potential for pharmacological development, in order to produce medicines for specific treatments related to digestive system disorders. Especially, we identify *Zingiber montanum,* but also to a lesser degree *Z. zerumbet* and *Z. ottensii*, as candidates for the treatment of flatulence (Table S2). These plants produce a range of terpenoids, flavonoids and alkaloids [32], the main secondary compounds being zerumbone and kaempferol derivatives [33]. Clinical trials, both in vitro and in vivo, found that the plants have antioxidant activities, anti-inflammatory activities, anti-allergic activities, hypotensive activities, antiarrhythmic activities, local analgesic and anesthetic activities, antibacterial and antifungal activities, and antihistaminic actions [32]. However, we still lack pharmacological evidence that the plants will be efficient in the treatments of flatulence, even if they are widely used for that purpose in many places including Thailand. Abdominal pain is another symptom which was often treated with gingers in Thailand. Our results suggests that eight ginger species might genuinely have potential as treatments for abdominal pain and peptic ulcers, respectively (Table S2). Phytochemical studies have proven that the plants contain high amounts of secondary metabolites, which are effective for various treatments, such as curcumin [34], zingiberene [35], gingerol [35] and zerumbone [36]. Several studies have provided scientific evidence that gingers have a broad variety of pharmacological activities. However, we rarely found evidence-based pharmacological research confirming that gingers have the potential for the treatment of abdominal pain, colitis, diarrhea, flatulence, laxative or peptic ulcers, except in studies of *Curcuma longa* L. Many modern medicines were developed from traditional uses [1]. An example is artemisinin [37]. Therefore, we recommend further research on the effect of ginger species in treating conditions related to digestive system disorders, especially for abdominal pain, colitis, diarrhea, flatulence, constipation and peptic ulcers. Specifically, we suggest the plant taxa from Table 3 and Table S2, which have high values for specific symptoms.

## 4. Materials and Methods

### 4.1. Ethnomedicinal Data

Thai traditional knowledge has a long history, and it is very difficult to find its origin. Traditionally, Thai traditional plant knowledge is communicated from the older generation to the younger orally. It is rarely found in text formats. The old literature, called *Tam Ra O-sod Phra Narai*, records Thai traditional knowledge, describing the medicinal use formularies of King Narai the Great’s reign (Ayutthaya period, 1656–1688). However, we have no evidence that the literature was recorded by the original healers or traditional physicians. Moreover, the literature was revised and published in several versions. In the present period, *Rattanakosin* (1782–present), a number of medicinal plant uses have been recorded in several forms. The old-fashioned notes are *Borassus* palm leaf booklets. Some notes were kept as clans’ secrets and passed to only one, or a few, of their heirs. In the present time, only a few of these ancient texts have been found, and the records mention plant names as local names, and sometimes in the old language. It is difficult to integrate this valuable old Thai traditional knowledge into the modern sciences. In the last few decades, medicinal plants have been popular among Thai people. Hundreds of books about medicinal plants have been published. Some of them mention scientific plant names with uses. However, as scientists, we cannot use them as references in this analysis. Our concerns are as follows: (1) The scientific plant names might be incorrect. Most authors are not botanists, so we cannot trust their plant identification process. In cases where they did not identify the species themselves, it is possible that they just got scientific names by researching the plants’ local names. (2) The sources of the knowledge are unspecified. The literature that specifies only plant descriptions and uses is useless for this study. It is possible that data from many books have the same sources. This will interfere with the study’s results, unexpectedly duplicating data. This study, therefore, focuses on only the sources of the literature that use ethnobotanical methods, explained in [38], for collecting data. The literature must provide the study location, scientific plant names (including identification methods), and uses. The ethnomedicinal reports of the gingers were extracted from 62 scientific publications, which were published between 1990 and 2019. The year 1990 was the first year that ethnobotany was introduced into the Thai academic system [39]. Although Thailand has a long history of taxonomic work, only rarely do herbarium specimen labels describe the uses of plants. Most herbarium specimens with plant use descriptions were prepared by ethnobotanists, and the data were published their works. Therefore, we included only data from ethnobotanical publications, and we ignored herbarium label data so as to avoid data duplication. The references included one book, 26 post-graduate theses, 32 journal articles and 3 scientific reports (Supplementary Materials). All these references used ethnobotanical methods to collect data. We avoided duplication by eliminating publications that were done by the same authors in the same place and at the same time. When the results were presented in the students’ thesis, we focused on the data from their thesis, and ignored the use reports from subsequent journal articles based on the same data. An example is the data from the thesis published by Srithi (2012) [39], which was published in parts in three journal articles [40,41,42]. In this case, we only included the data from the thesis. Data cited in a reference that were based on previous references were also eliminated. An example is that some data used in a thesis [43] were based on previous reports [44,45,46], and they were already published in three articles [47,48,49]. In this case, we used the data from the oldest reports. We screened the references from the online database of the Thai Library Integrated System [50], which collects all theses and academic reports from all higher educational institutions in Thailand. For additional data sources, we searched the Google Scholar, PubMed and Scopus databases. Moreover, we screened the publications in the Thai language from national journals. Therefore, we are confident that the references included in our study cover almost all ethnomedicinal use reports pertaining to gingers during the selected period, and we have made every effort to ensure the data are not repeated in our material. As the vernacular names of Thai plants are problematic and can cause confusion, we excluded all use reports without scientific plant names. In cases where the authors could only identify a plant to a genus (e.g., as *Curcuma* sp., *Alpinia* sp., *Zingiber* sp., etc.), these partly identified species were also excluded from our analysis. Because some references were old, we updated the plants’ scientific names following the World’s Check List of vascular plants.

Finally, each report was classified into a use category following the *International Classification of Primary Care* (ICPC-2). As the aim of this study is to find plant candidate taxa for future research, the definitions of sicknesses and symptoms must be clear. In general, some sickness or symptom names recognized by local healers are totally or partially different from the names of health conditions as they are in modern medical system. Based on the original data in the references and our field experiences, we carefully translated symptoms and sicknesses into medical terms. Lastly, those translated symptoms and sickness were matched with symptoms and sicknesses in the list of categories from ICPC-2 (Table 4).

### 4.2. Data Analysis

#### 4.2.1. Important Ginger Taxa

The relative importance of medicinal ginger taxa were calculated using the modified Cultural Importance Index (CI), the original version [51] of which is calculated as:

CI=UR/N

where UR is the total number of use reports for a species, and N is the total number of informants in the interview. As all the references used in this analysis used ethnobotanical methods in their studies, they specified the methods for collecting data very well, including locations, seasons, samplings and interviewing methods. Most of the studies used semi-structured interviews to collect data, while some of them used opened or structured interviews. As all these kinds of interviewing methods yield similar data for the uses of a single plant species, each use of a species from a reference refers to a single use report (UR) in this study. When using meta-data for analysis, it is difficult to connect a single use report to a single informant possessing the knowledge. We combined knowledge from all informants living in the same villages into one dataset. We use the term “pseudoinformant” to replace an individual person, “informant”, with a village from which the information is derived. This term is the same as the one we use in our previous study [24]. Hence, the CI values shown in this study were calculated from the following equation:

$$\mathrm{CI}=\mathrm{UR}/N_{\mathrm{ps}}$$

where N_ps_ is the number of pseudoinformants or the number of studied villages. A high CI value indicates that the plant is commonly known among pseudoinformants, i.e., among the study villages. On the other hand, CI values close to zero (0) show that the plant is rarely used.

#### 4.2.2. Informant Consensus Factor (ICF)

The medicinal categories treated by gingers were compared using the Informant Consensus Factor (ICF) [52], which is calculated as:

ICF=(Nur−Nt)/(Nur−1)

where Nur represents the number of use reports in a use category and Nt is the number of species with ethnomedicinal properties in that category. When ICF is equal to 1, it indicates that all informants agree on the use of the plant for a particular treatment. It also suggests that traditional knowledge has been exchanged among informants, or that the purpose of plant selection was well-defined. It may suggest the efficacy of the plant for the treatment. On the other hand, the lowest ICF value (equal to zero) suggests that informants do not agree on the use of a particular plant for the treatment, or that it is a randomly selected plant used as medicine.

## 5. Conclusions

Many medicines were developed from ethnomedicinal uses. Species in the ginger family are some of the most commonly used among Thai medicinal plants. The majority of studies on gingers focus on *Curcuma longa* L. Here, we document the large number of ethnomedicinal uses for the 76 ginger species in Thailand, and suggest additional species which might have potential applicability in the treatments, especially the *Zingiber* species. The result strongly suggests that the ICPC-2 standard can identify an important medicinal category, in the treatment of which gingers have therapeutic effect, especially for symptoms and health conditions related to digestive system disorders. We highlight the ginger genera and species that have potential for use in the treatment of health conditions, specifically for abdominal pain, colitis, diarrhea, flatulence, constipation and peptic ulcers. We hope that our results will encourage other scholars to use this information for pharmacological development, which can help people around the world.

## Figures and Tables

**Table 1:** Informant Consensus Factor (ICF) values for Thai gingers in each ICPC-2 category, as reported in 62 references.

Category	ICF
Digestive	0.86
General and unspecified	0.71
Skin	0.69
Pregnancy, childbearing, family planning	0.68
Male genital	0.67
Respiratory	0.64
Musculoskeletal	0.55
Neurological	0.51
Ear	0.50
Eye	0.50
Psychological	0.42
Cardiovascular	0.41
Endocrine/metabolic and nutritional	0.37
Urological	0.32
Female genital	0.31
Blood, blood forming organ and immune mechanism	0.17

**Table 2:** Informant Consensus Factor (ICF) values for uses of Thai gingers for the most commonly treated symptoms related to the ICPC-2 category of digestive system disorders.

Category	No. of Use Reports	ICF
Flatulence	111	0.70
Abdominal pain	77	0.67
Diarrhea	22	0.57
Peptic ulcers	27	0.50
Constipation	22	0.43
Colitis	21	0.40

**Table 3:** Cultural Importance Index (CI) for ginger genera used in the treatment of six health conditions under the category of digestive system disorders (abdominal pain, colitis, diarrhea, flatulence, constipation and peptic ulcers) extracted from 62 references covering 104 villages in Thailand.

Genus	CI Value of Disease/Symptom					
	Abdominal Pain	Colitis	Diarrhea	Flatulence	Constipation	Peptic Ulcers
*Alpinia*	0.03	0.02	0.27	0.08	0.32	1.24
*Amomum*	0.00	0.00	0.03	0.00	0.02	0.05
*Boesenbergia*	0.00	0.00	0.02	0.00	0.00	0.02
*Curcuma*	0.00	0.00	0.00	0.00	0.02	0.02
*Etlingera*	0.00	0.00	0.00	0.00	0.00	0.02
*Globba*	0.00	0.02	0.03	0.00	0.13	0.34
*Hedychium*	0.00	0.00	0.05	0.02	0.13	0.35
*Kaempferia*	0.02	0.00	0.18	0.05	0.65	1.79
*Stahlianthus*	0.00	0.00	0.06	0.00	0.00	0.10
*Zingiber*	0.00	0.00	0.00	0.00	0.02	0.02

**Table 4:** List of ICPC-2 categories linked to symptoms/treatments/sicknesses translated from ethnic symptoms/treatments/sicknesses from the Thai language.

ICPC-2 Category	Symptom/Treatment/Sickness	Ethnic Symptom/Treatment/Sickness (in Thai)
A: General and Unspecified	Anthelminthic	Kab Pa Yad
	Antidote	Kae Pid
	Antipyretic	Kae Kai
	Aphrodisiac	Ya Bam Rung Tang Ped
	Cancer	Ma Reng
	Chest oppression	Nan Na Ok
	Chronic fever	Kai Rua Rung
	Cold	Wad
	Faintness	Na Mued
	Fever	Kai
	Fever in children	Kai Nai Dek
	Flu-Like Syndrome	Kran Nua Kran Tua
	Hives	Rok Lom Pid
	Inflamation	Kae Ak Seb
	Internal body heat	Ron Nai
	Internal bruises	Cham Nai
	Oedema	Buam Nam
	Pain	Puad
	Plant poison antidote	Kae Pid Jak Pued
	Toothache	Puad Fan
	Tuberculosis	Wan Na Rok/Fee Nai Tong
	Veneral disease	Kam Ma Rok
	Wounds	Rak Sa Plae
	Headache	Puad Hua
B: Blood, Blood Forming Organs and Immune Mechanism	Hemostatic	Ya Ham Luad
	Stop bleeding	Ham Luad
D: Digestive	Abdominal pain	Puad Tong
	Antiemetic	Kae A-Jean
	Apthous ulcer	Ron Nai
	Carminative	Kab Lom
	Colitis	Lam Sai Ak Seb
	Constipation	Tong Pook
	Diarrhea	Tong Sea
	Dysentery	Bid
	Flatulence	Tong Ued
	Food poisoning	A-harn Pen Pid
	Gastric ulcer	Plae Nai Kra Po
	Gastritis	Rok Kra Po
	Hepatopathy	Rok Tub
	Hernia	Sai Luan
	Indigestion	A-harn Mai Yoi
	Jaundice	Dee San
	Laxative	Ya Ra Bai
	Nausea	Kluen Sai
	Stomach ache	Puad Tong
F: Eye	Conjunctivitis	Ta Daeng
	Eye ache	Puad Ta
	Ophthalmitis	Yua Ta Ak Seb
	Trachoma	Rid See Duang Ta
H: Ear	Earache	Puad Hoo
	Otitis	Hoo Ak Seb
	Otorrhea	Hoo Nam Nuak
K: Cardiovascular	Congestion	Luad Kung
	Heart problems	Rok Hua Jai
	Hemorrhoids	Rid See Duang
	Hypertension	Kwam Dan Lo Hit Soong
	Increase blood flow	Chuay Hai Luad Lom Lai Wian Dee
	Promote blood circuation	Kra Tun Kan Lai Wian Kong Luad
L: Musculoskeletal	Back and waist pain	Puad Lung/Puad Ew
	Bone and joint pain	Puad Koh Puad Kra Dook
	Bone symptoms	Rok Kra Dook
	Cramp	Ta Crew
	Discomfort muscle	Puad Muay
	Dislocation	Kra Dook Lud
	Fractures	Kra Dook Huk
	Knee pain	Puad Kao
	Muscular relaxation	Ya Clai Klam Nue
	Osteoarthritis	Rok Ko Suam
	Sprain	Kae Kled
N: Neurological	Anaesthesia	Tam Hai Cha
	Convulsion	Sun
	Dizziness	Wing Wian
	Paralysis	Am Ma Pad
R: Respiratory	Nosebleed	Luad Kam Dao Lai
	Asthma	Huad
	Cough	Ai
	Expectorant	Kub Saem Ha
	Halitosis	Pa Wa Mee Klin Pak
	Nasal polyp	Rid See Duang Cha Mook
	Sore throat	Kae Cheb Kow
	Stuffed nose	Kad Cha Mook
S: Skin	Athlete’s foot	Nam Kad Tao
	Blister	Plae Pu Pong
	Bruised	Plae Fok Cham
	Burns	Plae Fai Mai/Nam Ron Luak
	Canker	Pak Puay
	Catepillar allergy	Pae Non/Bung
	Chronic rash	Kan Rua Rung
	Dandruff	Rung Kae
	Dermatophytosis	Klak Kluan
	Dermatosis	Rok Pew Nung
	Herpes	Ngoo Sa Wad
	Insect bites and stings	Kae Malaeng Sad Kad Toi
	Insect repellant	Ya Lai Malaeng
	Pruritus	A-Karn Kan
	Pus	Nong
	Ringworm	Klak
	Skin disease	Rok Pew Nung
	Snake bites	Kae Ngoo Kad
	Umbilical wound	Plae Tee Sa Due
	Venomous animal bites	Kae Pid Sad
	Zoster	Ngoo Sa Wad
T: Endocrine/ Metabolic and Nutritional	Appetite stimulant	Chuay Cha Roen A-harn
	Beriberi	Neb Cha
	Bile tonic	Bam Rung Nam Dee
	Blood tonic	Bam Rung Luad
	Brain tonic	Bam Rung Samong
	Cardiotonic	Bam Rung Hua Jai
	Diabetes mellitus	Bao Wan
	Gout	Rok Gout
	Haematonic	Ya Bam Rung Luad
	Skin nourishment	Bam Rung Pew Nung
	Tonic	Ya Bam Rung
	Wasting disease	Rok Pom Haeng
	Women’s tonic	Ya Bam Rung Ped Ying
	Men’s tonic	Bam Rung Ped Chai
U: Urological	Anuria	Pad Sa Wa Kad
	Diuretic Agents	Kub Pud Sa Wa
	Nephritis	Tai Ak Seb
	Urinary stones	New
W: Pregnancy, Childbearing, Family Planning	Amniotic fluid elimination	Kab Nam Kaw Pla
	Contraceptive	Kum Kam Nerd
	Galactagogue	Ya Puem Nam Nom
	Postpartum abdominal pain	Puad Tong Lung Klod
	Postpartum convulsion	Kae A-Karn Sun Lung Klod
	Postpartum nervous	Lom Pid Duan
	Postpartum tonic	Bam Rung Lung Klod
X: Female Genital	Amenorrhoea	Kad Ra Doo
	Dysmenorrhea	Puad Pra Cham Duan
	Haemagogue	Ya Kab Ra Doo
	Irregular menstruation	Pra Cham Duen Mai Pa Ka Ti
	Leucorrhea	Tok Kaw
	Uterine involution	Chuay Hai Mod Look Kao Oo

## Supplementary Materials

The following are available online at https://www.mdpi.com/2223-7747/9/7/906/s1, Table S1: Ethnomedicinal uses of gingers in Thailand; Table S2: Fidelity Level (FL) of gingers for treatments of some symptoms in Digestive system disorders.
