# Supplementary material for: Using ICPC-2 Standard to Identify Thai Zingiberaceae of Pharmacological Interest
Source: Plants (Basel). 2020 Jul 17;9(7):906. doi: 10.3390/plants9070906 (PMC7412399; doi:10.3390/plants9070906)
Supplement: Supplementary file 1 [file plants-09-00906-s001.pdf]

Supplementary Materials

Thai Zingiberaceae of pharmacological interest

Phumthum, M. and Balslev, H.

Table S1 Ethnomedicinal uses of gingers in Thailand.

Plant Name	Uses (Preparation/Application)	References
Alpinia		
Alpinia calcarata (Haw.) Roscoe	Diarrhea (dc/oi), diuretic agents (dc/oi)	Anderson (1993), Srithi (2012)
Alpinia conchigera Griff.	Asthma (cr/al), diabetes mellitus (un/un)	Chuakul et al. (2004), Chuakul and Boonpleng (2003)
Alpinia galanga (L.) Willd.	Amenorrhoea (un/un), anaesthesia (sw/ba), anthelminthic (un/un), back and waist pain (dc/oi), carminative (un/un), colitis (cf/oi), congestion (un/un), convulsion (un/un), cough (dc/oi), dermatophytosis (un/un), diarrhea (np/oi), dizziness (cr/sm), fever (dc/oi), flatulence (dc/ba), headache (dc/oi), hypertension (un/un), insect bites and stings (dc/oi), laxative (un/un) muscular relaxation (un/un), paralysis (un/un), peptic ulcers (un/un), postpartum abdominal pain (un/un), postpartum nervous (dc/ba), postpartum tonic (un/un), toothache (dc/oi), venomous animal bites (cr/al)	Chuakul (2012), Chuakul and Boonpleng (2003), Chuakul et al. (2002a), Gunsuwan (2011), Inta (2008), Jitjum et al. (2019), Kaewwongsiri and Saiyapan (2004), Khonkayan et al. (2019), Khuankaew (2014), Neamsuvan (2013a), Nuammee (2012), Pannet (2008), Panyadee et al. (2019), Ponpim (1996), Pantarod (2002), Purintavaragul et al. (2012), Sonsupub (2010), Srithi (2012), Thaenkam et al. (2019), Winijchaiyanan (1995)
Alpinia malaccensis (Burm.f.) Roscoe	Abdominal pain (np/oi), cough (dc/oi), dermatophytosis (po/un), flatulence (np/oi), food poisoning (np/oi), indigestion (dc/oi), pruritus (dc/oi), stomach ache (dc/oi), tuberculosis (dc/oi)	Anderson (1993), Kaewsangsai (2017), Kantasrila (2016), Neamsuvan et al. (2014), Neamsuvan and Tuntien (2015), Panyadee et al. (2019), Srisanga et al. (2011), Sukkho (2008)
Alpinia mutica Roxb.	Tonic (un/un), urinary stones (un/un)	Chuakul and Boonpleng (2003), Upho (2005)
Alpinia nigra (Gaertn.) Burtt	Chronic rash (un/un), fever (un/un), stop bleeding (un/un), tonic (un/un), wounds (un/al)	Charnprasert and Chansuwanit (1992), Chuakul and Boonpleng (2003)
Alpinia oxymitra K.Schum.	Tonic (un/un)	Chuakul and Boonpleng (2003)
Alpinia roxburghii Sweet	Dysmenorrhea (un/un), haematonic (un/un)	Chuakul and Boonpleng (2003), Kaewsangsai (2017), Kantasrila (2016)
Alpinia siamensis K.Schum.	Dermatophytosis (po/un), internal bruises (dc/un), muscular relaxation (dc/ba)	Wayo (2012)
Alpinia zerumbet (Pers.) B.L.Burtt & R.M.Sm.	Anthelminthic (sw/oi), cold (dc/oi), cough (dc/oi), diarrhea (un/un), earache (un/al), faintness (pd/oi), fever (dc/oi), flu-like syndrome (dc/oi), hives (dc/ba),	Anderson (1993), Chuakul (2012), Chuakul and Boonpleng (2003), Chuakul et al. (2002a), Kaewwongsiri and Saiyapan (2004), Neamsuvan and

	paralysis (un/un), peptic ulcers (dc/oi), tonic (un/un)	Tuntien (2015), Nuammee (2012), Panyaphu (2012), Srithi (2012), Tangtragoon (1998), Yaso (2000)
Amomum		
Amomum curtisii (Baker) Skornick. & Hlavatá	Abdominal pain (un/un), flatulence (un/un)	Chuakul and Boonpleng (2003) Upho (2005)
Amomum dealbatum Roxb.	Abdominal pain (un/un), colitis (un/un), cough (dc/oi)	Inta (2008), Nuammee (2012), Srithi (2012)
Amomum siamense Craib	Muscle discomfort (un/un)	Anderson (1993)
Amomum trilobum Gagnep.	Haematonic (un/un)	Chuakul and Boonpleng (2003)
Boesenbergia		
Boesenbergia longiflora (Wall.) Kuntze	Tonic (un/un)	Chuakul and Boonpleng (2003)
Boesenbergia plicata (Ridl.) Holttum	Cardiotonic (un/un), fever (un/un), flatulence, tonic (un/un)	Juntarapol et al (2014), Thaenkam et al. (2019), Upho (2005)
Boesenbergia rotunda (L.) Mansf.	Anuria (un/un), canker (un/un), cardiotonic (dc/oi), carminative (un/un) colitis (un/un), diuretic agents (un/un), dysentery (un/un), flatulence (dc/oi), galactagogue (un/un), haemagogue (un/un), halitosis (un/un), oedema (un/un), paralysis (un/un), sexual stimulant (un/un), tonic (un/un) urinary polyp (un/un), wasting disease (un/un)	Chuakul and Boonpleng (2003), Junkhonkaen (2012), Khonkayan et al. (2019), Khuankaew (2014), Neamsuvan (2013a), Neamsuvan (2013b), Neamsuvan et al. (2012), Sinworn and Viriyawattana (2014), Sonsupub (2010), Upho (2005), Winijchaiyanan (1995)
Boesenbergia xiphostachya (Gagnep.) Loes.	Flatulence (un/un), laxative (un/un)	Chuakul and Boonpleng (2003)
Curcuma		
Curcuma aeruginosa Roxb.	Abdominal pain (dc/oi), convulsion (un/un), cough (un/un), dysentery (po/oi), flatulence (dc/oi), headache (dc/oi), muscular relaxation (dc/oi), peptic ulcers (un/un), postpartum injuries (un/un), postpartum tonic (un/un), snake bites (dc/un), stomach ache (dc/oi), wounds (po/al)	Anderson (1993), Nuammee (2012), Phueaknang (2005), Srithi (2012), Sukkho (2008), Tichachart (2004)
Curcuma amarissima Roscoe	Abdominal pain (pd/oi)	Muangyen (2013)
Curcuma angustifolia Roxb.	Pruritus (po/al)	Sukkho (2008)
Curcuma aurantiaca Zijp	Hemostatic (un/un)	Chuakul and Boonpleng (2003)
Curcuma comosa Roxb.	Abdominal pain (un/un), amniotic fluid elimination (dc/oi), flatulence (dc/oi) hernia (un/un), irregular menstruation (dc/oi), muscular relaxation (dc/oi), oedema (dc/oi), peptic ulcers (un/un), postpartum injuries (un/un), postpartum tonic (un/un), uterine involution (un/un), women's tonic (dc/oi)	Chuakul and Boonpleng (2003), Imchan (2006), Neamsuvan et al. (2012), Nuammee (2012), Purintavaragul et al. (2012), Srithi (2012)
Curcuma euchroma Valeton	Peptic ulcers (un/oi)	Panyaphu (2012)

Curcuma involucrata (King ex Baker) Kornick.	Abdominal pain (un/un), anuria (un/un), constipation (un/un), diarrhea (un/un), flatulence (np/oi), internal body heat (dc/oi), sprain (dc/oi)	Anderson (1993), Nuammee (2012), Srithi (2012)
Curcuma longa L.	Abdominal pain (np/oi), abdominal pain (un/oi), bile tonic (un/un), bruised (po/ms), carminative (dc/oi), colitis (un/un), dermatosis (un/un), fever (dc/oi), flatulence (dc/oi), flu-like syndrome (cr/al), gastric ulcer (np/oi) gastritis (dc/oi), haemagogue (un/un) indigestion (un/un), intestinal infection (un/un), laxative (dc/oi), paralysis (un/un), peptic ulcers (un/un), postpartum tonic (un/st), pruritus (dc/un), sore throat (np/oi), sprain (hc/ms, stomach ache (dc/oi), tonic (dc/oi), urinary stones (hc/un), venomous animal bites (un/un), wounds (dc/al)	Anderson (1993), Chuakul (2005), Chuakul (2012), Chuakul and Boonpleng (2003), Chuakul et al. (2002a), Imchan (2006), Inta (2008), Junkhonkaen (2012), Neamsuvan and Tuntien (2015), Neamsuvan et al. (2012), Nuammee (2012), Pannet (2008), Panyadee et al. (2019), Ponpim (1996), Poonthananiwatkul et al. (2015), Purintavaragul et al. (2012), Siriyong et al. (2019), Srithi (2012), Sukkho (2008), tangjitman (xxxx), Tovaranonte (1998), Wayo (2012), Winijchaiyanan (1995), Yaso (2000)
Curcuma mangga Valeton & Zijp	Anthelminthic (un/un), carminative (un/un), colitis (dc/oi), urinary stones (un/un)	Noitana (2013), Phueaknang (2005), Sonsupub (2010)
Curcuma oligantha Trimen	Tonic (un/un)	Chuakul and Boonpleng (2003)
Curcuma parviflora Wall.	Flatulence (dc/oi), flu-like syndrome (dc/oi), stop bleeding (un/un), wounds (un/al)	Anderson (1993), Inta et al. (2013), Neamsuvan and Tuntien (2015), Sinworn and Viriyawattana (2014)
Curcuma sessilis Gage	Carminative (un/un), flatulence (dc/oi), laxative (un/un), pruritus (sw/ba), tonic (un/un), wounds (un/un)	Chuakul and Boonpleng (2003), Jearanai (2012), Ponpim (1996), Thaenkam et al. (2019), Winijchaiyanan (1995)
*Curcuma sparganii*folia Gagnep.	Asthma (dc/oi)	Chuakul and Boonpleng (2003), Chuakul et al. (2004)
Curcuma zanthorrhiza Roxb.	Amniotic fluid elimination (un/un), faintness (un/un), hemorrhoids (un/un), indigestion (un/un), irregular, menstruation (un/un), postpartum tonic (un/un)	Jearanai (2012), Phueaknang (2005)
Curcuma zedoaria (Christm.) Roscoe	Abdominal pain (un/un), blisters (un/un), chest oppression (un/un), colitis (un/un), congestion (un/un), dermatosis (un/un), dislocation (un/un), dysentery (dc/oi), fever (un/un), flatulence (dc/oi), headache (un/un), herpes (un/un), indigestion (un/un), loss of conciousness (dc/un), nasal polyp (un/un), paralysis (un/un), peptic ulcers (un/un), pruritus (un/un), wounds (dc/al)	Anderson (1993), Inta (2008), Junkhonkaen (2012), Kesornmas et al. (2019), Khonkayan et al. (2019), Neamsuvan (2013a), Neamsuvan (2013b), Neamsuvan et al. (2012), Neamsuvan et al. (2018), Pantarod (2002), Srithi (2012), Tovaranonte (1998)
Etlingera		
Etlingera elatior (Jack) R.M.Sm.	Anthelminthic (un/un), carminative (dc/oi), flatulence (un/oi), hypertension	Chuakul (2005), Junkhonkaen (2012), Srithi (2012), Upho (2005)

	(un/un), paralysis (pd/un), stomach ache (dc/oi)	
Etlingera littoralis (J.Koenig) Giseke	Anthelminthic (un/un), carminative (dc/oi), flatulence (un/oi), hypertension (un/un), paralysis (pd/un), stomach ache (dc/oi)	Chuakul (2005), Chuakul and Boonpleng (2003), Chuakul and Boonpleng (2003), Kaewwongsiri and Saiyapan (2004)
Etlingera megalocheilos (Griff.) A.D.Poulsen	Heart problems (dc/oi), tonic (dc/oi)	Anderson (1993), Srithi (2012)
Etlingera punicea (Roxb.) R.M.Sm.	Cardiotonic (pd/un), carminative (pd/un), stomachic (pd/un)	Chuakul (2005), Chuakul and Boonpleng (2003)
Gagnepainia		
Gagnepainia thoreliana (Baill.) K.Schum.	Hemostatic (pd/al), wounds (un/un)	Chuakul et al. (2004), Chuakul and Boonpleng (2003)
Globba		
Globba candida Gagnep.	Otitis (cr/al), otorrhea (un/un)	Chuakul et al. (2004), Chuakul and Boonpleng (2003)
Globba geoffrayi Gagnep.	Asthma (un/un)	Chuakul et al. (2004), Chuakul and Boonpleng (2003)
Globba laeta K.Larsen	Abdominal pain (dc/oi), flatulence (dc/oi), flu-like syndrome (dc/oi)	Imchan (2006), Neamsuvan and Tuntien (2015)
Globba obscura K.Larsen	Anuria (dc/oi), stomach ache (un/un), wounds (un/un)	Chuakul and Boonpleng (2003), Inta et al. (2013)
Globba winitii C.H.Wright	Flu-like syndrome (dc/oi)	Neamsuvan and Tuntien (2015)
Hedychium		
Hedychium coccineum Buch.-Ham. ex Sm.	Women's tonic (un/un)	Srithi (2012)
Hedychium coronarium J.Koenig	Cough (un/un), headache (dc/oi), muscular relaxation (dc/oi), sprain (un/ba), tonic (un/un), tuberculosis (dc/oi)	Anderson (1993), Chaunchom (2011), Chuakul and Boonpleng (2003), Ponpim (1996), Srisanga et al. (2011)
Hedychium ellipticum Buch.-Ham. ex Sm.	Abdominal pain (un/un), muscular relaxation (un/un)	Upho (2005)
Hedychium flavum Roxb.	Back and waist pain (un/un), bruised (un/un), postpartum tonic (un/un)	Srithi (2012), Winijchaiyanan (1995)
Hedychium stenopetalum Lodd.	Colitis (un/un), constipation (un/un)	Pantarod (2002)
Kaempferia		
Kaempferia elegans (Wall.) Baker	Fever (un/un), flatulence (un/un)	Chuakul and Boonpleng (2003), Kesornmas et al. (2019),
Kaempferia filifolia K.Larsen	Leucorrhea (dc/oi)	Chuakul et al. (2004), Chuakul and Boonpleng (2003)
Kaempferia galanga L.	Abdominal pain (dc/oi), antipyretic (dc/un), anuria (un/un), carminative (dc/oi), cold (un/un), colitis (dc/oi), colitis (un/un), congestion (un/un), constipation (un/un), cough (dc/oi), dandruff (un/un), diarrhea (dc/oi), expectorant (dc/oi), fever (dc/oi), flatulence (un/un), food poisoning (un/un), hives (dc/oi), insect bites and stings (un/un), nosebleed (un/un),	Chuakul (2005), Chuakul (2012), Chuakul and Boonpleng (2003), Chuakul et al. (2002a), Imchan (2006), Inta (2008), Junkhonkaen (2012), Neamsuvan et al. (2012), Noitana (2013), Nuammee (2012), Panyadee et al. (2019), Phueaknang (2005), Ponpim (1996), Sinworn and Viriyawattana (2014),Srisanga et al. (2011), Srithi

	oedema (un/un), ophthalmitis (un/un), paralysis (un/un), plant poison (un/un), sore throat (un/un), stuffed nose (po/un), tonic (dc/un), urinary stones (dc/un), urinary stones (un/un), wounds (un/al)	(2012), Sukkho (2008), Wannasri and Buapan (2013), Yaso (2000)
Kaempferia larsenii Sirirugsa	Antidote (un/un)<br>Insect bites and stings (pd/al)	Chuakul and Boonpleng (2003), Chuakul et al. (2004)
Kaempferia parviflora Wall. ex Baker	Abdominal pain (un/oi), anuria (un/un), aphrodisiac (dc/oi), blood tonic (un/oi), bruised (un/un), cardiotonic (un/un), cough (un/un), diarrhea (np/oi), dizziness (un/un), flatulence (dc/oi), food poisoning (dc/oi), gastritis (dc/oi), gout (un/un), laxative (un/un), men's tonic (dc/oi), paralysis (dc/oi), peptic ulcers (dc/oi), peptic ulcers (un/un), tonic (dc/oi), tuberculosis (dc/oi), wounds (pd/al)	Anderson (1993), Gunsuwan (2011), Imchan (2006), Inta (2008), Jearanai (2012), Junkhonkaen (2012), Kesornmas et al. (2019), Neamsuvan et al. (2012), Nuammee (2012), Panyaphu (2012), Phueaknang (2005), Srisanga et al. (2011), Srithi (2012), Sukkho (2008), Tovaranonte (1998)
Kaempferia roscoeana Wall.	Antidote (un/un), fever (dc/oi)	Chuakul and Boonpleng (2003), Inta et al. (2013b)
Kaempferia rotunda L.	Abdominal pain (un/un), antidote (un/un), back and waist pain (un/un), blood tonic (un/un), bruised (un/un), burns (un/un), diarrhea (un/un), flatulence (un/oi), laxative (dc/oi), muscular relaxation (un/un), nausea (dc/oi), peptic ulcers (un/un), stomach ache (dc/oi), stop bleeding (un/un), wounds (un/un)	Anderson (1993), Chuakul and Boonpleng (2003), Nuammee (2012), Srithi (2012)
Rhynchanthus		
Rhynchanthus longiflorus Hook.f.	Convulsion (dc/oi), hypertension (dc/oi)	Srisanga et al. (2011)
Scaphochlamys		
Scaphochlamys biloba (Ridl.) Holttum	Contraceptive (un/un)	Upho (2005)
Stahlianthus campanulatus Kuntze	Abdominal pain (np/oi), abdominal pain (un/un), anuria (un/un), diarrhea (un/un), flatulence (un/un), peptic ulcers (un/un)	Imchan (2006), Srithi (2012)
Wurfbainia		
Wurfbainia biflora (Jack) Skornick. & A.D.Poulsen	Bone and joint pain (un/un)	Srithi (2012)
Wurfbainia testacea (Ridl.) Skornick. & A.D.Poulsen	Asthma (dc/oi), carminative (un/un), colitis (un/oi), flatulence (dc/oi), flatulence (un/un), increase blood flow (dc/oi), irregular menstruation (dc/oi)	Chuakul and Boonpleng (2003), Junkhonkaen (2012), Neamsuvan et al. (2012), Siriyong et al. (2019)
Wurfbainia uliginosa (J.Koenig) Giseke	Carminative (un/un), peptic ulcers (dc/oi), pruritus (un/un), stomach ache (un/un)	Chuakul and Boonpleng (2003), Chuakul et al. (2002b), Phueaknang (2005)

Wurfbainia vera (Blackw.) Skornick. & A.D.Poulsen	Paralysis (un/un), zoster (pd/al)	Neamsuvan (2013a), Kaewwongsiri and Saiyapan (2004)
Wurfbainia villosa (Lour.) Skornick. & A.D.Poulsen	Anuria (un/un), carminative (un/un), paralysis (un/un), stomach ache (un/un)	Khonkayan et al. (2019), Neamsuvan (2013a), Neamsuvan et al. (2018) Chuakul and Boonpleng (2003), Junkhonkaen (2012)
Zingiber		
Zingiber chrysostachys Ridl.	Tonic (un/un)	Chuakul and Boonpleng (2003)
Zingiber kerrii Craib	Carminative (dc/oi), flatulence (un/oi)	Inta et al. (2013)
Zingiber latifolium Theilade & Mood	Fractures (dc/oi), tonic (dc/oi)	Sukkho (2008)
Zingiber montanum (J.Koenig) Link ex A.Dietr.	Abdominal pain (dc/oi), amniotic fluid elimination (dc/oi), anthelminthic (un/un), apthous ulcer (dc/oi), athlete's foot (cr/al), beriberi (un/un), bone and joint pain (po/al), bone symptoms (un/un) bruised (un/un), carminative (dc/oi), catepillar allergy (un/un), cold (un/un), colitis (un/un), congestion (un/un), conjunctivitis (hc/un), constipation (un/un), cough (un/un), dermatosis (po/un), diarrhea (un/un), eye pain (hc/un), fever (dc/ba), fever in children (un/un), flatulence (dc/oi), flu-like syndrome (cr/smell), fractures (po/un), gastric ulcer (dc/oi), hepatopathy (un/un), herpes (un/un) hives (un/un), insect bites and stings (po/al), insect repellant (un/un), jaundice (np/un), laxative (dc/oi), muscular relaxation (hc/ms), oedema (dc/oi) pain (dc/oi), paralysis (un/un), peptic ulcers (un/un), postpartum bath (dc/ba), postpartum haemagogue (un/un), postpartum injuries (un/un) ,postpartum tonic (dc/ba), postpartum tonic (dc/oi), pruritus (un/un), skin nourishment (un/un), sprain (hc/un), urinary stones (un/un), veneral disease (un/un) wounds (un/al)	Anderson (1993), Chuakul and Boonpleng (2003), Chuakul et al. (2002a), Imchan (2006), Inta (2008), Inta et al. (2013), Inta et al. (2013b), Jitjum et al. (2019), Junkhonkaen (2012), Kesornmas et al. (2019), Khuankaew (2014), Manenoon et al. (2017), Muangyen (2013), Neamsuvan (2013a), Neamsuvan and Tuntien (2015), Neamsuvan et al. (2012), Nuammee (2012), Pannet (2008), Pantarod (2002), Panyadee et al. (2019), Phueaknang (2005), Ponpim (1996), Purintavaragul et al. (2012), Sinworn and Viriyawattana (2014), Sonsupub (2010), Srithi (2012), Sukkho (2008), Suwanantawong (2011), tangjitman (xxxx), Tangtragoon (1998), Thangthaisong et al. (2011), Tovaranonte (1998), Trisonthi (XXXX), Upho (2005), Wayo (2012), Winijchaiyanan (1995)
Zingiber niveum Mood & Theilade	Flatulence (dc/oi), laxative (dc/oi), paralysis (un/un)	Chuakul and Boonpleng (2003), Chuakul et al. (2004), Thangthaisong et al. (2011)
Zingiber officinale Roscoe	Antiemetic (np/un), appetite stimulant (dc/oi), backache (dc/oi) colitis (dc/oi), convulsion (un/un), cough (dc/oi) , expectorant (np/un), fever (dc/oi), flatulence (dc/oi), headache (un/un), hemorrhoids (un/un), mouth polyp	Amorndoljai (2018), Anderson (1993), Chuakul and Boonpleng (2003), Chuakul et al. (2002a), Chuakul et al. (2006), Gunsuwan (2011), Imchan (2006), Junkhonkaen (2012), Kesornmas et al. (2019), Neamsuvan (2013a), Neamsuvan (2013b), Neamsuvan et al. (2012), Neamsuvan et al. (2018), Pannet

	(un/un), muscular relaxation (po/ms), nasal polyp (un/un), oedema (un/un) paralysis (un/un), postpartum, convulsion (un/un), postpartum tonic (un/un), pus (po/un), sore throat (un/un) tonic (dc/oi), trachoma (un/un), urinary, polyp (un/un), veneral disease (un/un), venomous animal bites (un/un), wounds (hc/ms)	(2008), Pantarod (2002), Panyadee et al. (2019), Siriyong et al. (2019) Sonsupub (2010), Srisanga et al. (2011), Srithi (2012), Sukkho (2008), Thaenkam et al. (2019), Tovaranonte (1998), Wayo (2012), Winijchaiyanan (1995), Yaso (2000)
Zingiber ottensii Valeton	Abdominal pain (dc/oi), back and waist pain (un/un), caterpillar allergy (cr/un), colitis (un/un), diarrhea (dc/oi), flatulence (dc/oi), headache (po/ms), jaundice (un/un), laxative (dc/oi), muscular relaxation (po/ms), numbness (un/un), postpartum tonic (dc/oi), pruritus (po/ms), sore throat (po/ms), umbilical wound (np/oi)	Chuakul and Boonpleng (2003), Khonkayan et al. (2019), Nuammee (2012), Pannet (2008), Panyadee et al. (2019), Pipitkul (2001), Srithi (2012), Sukkho (2008), Upho (2005)
Zingiber rubens Roxb.	Stomach ache (un/un)	Anderson (1993)
Zingiber smilesianum Craib	Flatulence (un/oi)	Khuankaew (2014)
Zingiber spectabile Griff.	Tonic (un/un)	Chuakul and Boonpleng (2003)
Zingiber wrayi Prain ex Ridl.	Flu-like syndrome (dc/oi), galactagogue (un/un)	Chuakul and Boonpleng (2003), Neamsuvan and Tuntien (2015)
Zingiber zerumbet (L.) Roscoe ex Sm.	Amniotic fluid elimination (dc/oi), anuria (un/un), appetite stimulant (un/un), carminative (cr/oi), chest oppression (un/un), chronic fever (un/un), colitis (dc/oi), chronic fever (dc/oi), dysentery (dc/oi), expectorant (un/un), faintness (dc/oi), flatulence (dc/oi), galactagogue (dc/oi), haemagogue (dc/oi), indigestion (dc/oi, inflammation (un/un), laxative (dc/oi), muscular relaxation (dc/oi), paralysis (un/un), tonic (dc/oi)	Chuakul and Boonpleng (2003), Inta et al. (2013b), Kaewwongsiri and Saiyapan (2004), Khuankaew (2014), Neamsuvan (2013a), Neamsuvan et al. (2014), Neamsuvan and Tuntien (2015), Neamsuvan et al. (2012), Purintavaragul et al. (2012), Satean et al. (2018), Sinworn and Viriyawattana (2014), Sonsupub (2010)

al = applied locally, ba = bath, cr = crushed, cf = cooked as food, dc = decoction, hc = made a herbal compress, ms = massage, np = non-prepared, oi = oral ingestion, po = pounded, pd = powder, sm= smell, sw = soaked in water, un = unspecified

Table S2 Fidelity Level (FL) of gingers for treatments of some symptoms in Digestive system disorders.

**Species**	**Abdominal Pain**	**Colitis**	**Diarrhea**	**Flatulence**	**Laxative**	**Peptic Ulcers**
Alpinia calcarata (Haw.) Roscoe	0.00	0.00	0.03	0.00	0.00	0.00
Alpinia conchigera Griff.	0.00	0.00	0.00	0.00	0.00	0.00
Alpinia galanga (L.) Willd.	0.00	0.03	0.03	0.04	0.01	0.01
Alpinia malaccensis (Burm.f.) Roscoe	0.03	0.00	0.00	0.03	0.00	0.00

Alpinia mutica Roxb.	0.00	0.00	0.00	0.00	0.00	0.00
Alpinia roxburghii Sweet	0.00	0.00	0.00	0.00	0.00	0.00
Alpinia zerumbet (Pers.) B.L.Burtt & R.M.Sm.	0.00	0.00	0.01	0.00	0.00	0.03
Amomum dealbatum Roxb.	0.01	0.01	0.00	0.00	0.00	0.00
Amomum testaceum Ridl.	0.00	0.01	0.00	0.04	0.00	0.00
Amomum uliginosum J.Koenig	0.01	0.00	0.00	0.01	0.00	0.01
Amomum villosum Lour.	0.01	0.00	0.00	0.01	0.00	0.00
Boesenbergia rotunda (L.) Mansf.	0.00	0.01	0.00	0.07	0.00	0.00
Boesenbergia xiphostachya (Gagnep.) Loes.	0.00	0.00	0.00	0.01	0.01	0.00
Curcuma aeruginosa Roxb.	0.09	0.00	0.00	0.04	0.00	0.03
Curcuma amarissima Roscoe	0.01	0.00	0.00	0.00	0.00	0.00
Curcuma comosa Roxb.	0.01	0.00	0.00	0.01	0.00	0.01
Curcuma euchroma Valeton	0.00	0.00	0.00	0.00	0.00	0.01
Curcuma longa L.	0.05	0.04	0.00	0.07	0.03	0.11
Curcuma mangga Valeton & Zijp	0.00	0.01	0.00	0.01	0.00	0.00
Curcuma parviflora Wall.	0.00	0.00	0.00	0.01	0.00	0.00
Curcuma sessilis Gage	0.00	0.00	0.00	0.04	0.01	0.00
Curcuma zanthorrhiza Roxb.	0.00	0.00	0.00	0.00	0.00	0.00
Curcuma zedoaria (Christm.) Roscoe	0.01	0.01	0.00	0.01	0.00	0.01
Elettariopsis curtisii Baker	0.00	0.00	0.00	0.01	0.00	0.00
Elettariopsis curtisii Baker	0.03	0.00	0.00	0.03	0.00	0.00
Etlingera elatior (Jack) R.M.Sm.	0.01	0.00	0.00	0.03	0.00	0.00
Etlingera littoralis (J.Koenig) Giseke	0.00	0.00	0.00	0.03	0.00	0.01
Etlingera punicea (Roxb.) R.M.Sm.	0.00	0.00	0.00	0.03	0.00	0.00
Globba laeta K.Larsen	0.01	0.00	0.00	0.01	0.00	0.00
Globba obscura K.Larsen	0.01	0.00	0.00	0.00	0.00	0.00
Hedychium coccineum Buch.-Ham. ex Sm.	0.00	0.00	0.00	0.00	0.00	0.00
Hedychium ellipticum Buch.-Ham. ex Sm.	0.01	0.00	0.00	0.00	0.00	0.00
Hedychium stenopetalum Lodd.	0.00	0.01	0.00	0.00	0.00	0.00
Kaempferia elegans (Wall.) Baker	0.00	0.00	0.00	0.01	0.00	0.00
Kaempferia filifolia K.Larsen	0.00	0.00	0.00	0.00	0.00	0.00
Kaempferia galanga L.	0.07	0.04	0.01	0.04	0.00	0.00
Kaempferia parviflora Wall. ex Baker	0.07	0.00	0.03	0.05	0.01	0.05
Kaempferia rotunda L.	0.09	0.00	0.01	0.01	0.03	0.01
*Stahlianthus camp*anulatus Kuntze	0.07	0.00	0.01	0.04	0.00	0.01
*Stahlianthus involu*cratus (King ex Baker) Craib ex Loes.	0.04	0.00	0.03	0.01	0.00	0.00
Zingiber kerrii Craib	0.00	0.00	0.00	0.03	0.00	0.00
Zingiber montanum (J.Koenig) Link ex A.Dietr.	0.11	0.04	0.04	0.20	0.01	0.03
Zingiber niveum Mood & Theilade	0.00	0.00	0.00	0.01	0.03	0.00
*Zingiber offi*cinale Roscoe	0.00	0.01	0.00	0.04	0.00	0.00
Zingiber ottensii Valeton	0.11	0.03	0.04	0.08	0.07	0.00

Zingiber rubens Roxb.	0.01	0.00	0.00	0.00	0.00	0.00
Zingiber smilesianum Craib	0.00	0.00	0.00	0.01	0.00	0.00
Zingiber zerumbet (L.) Roscoe ex Sm.	0.00	0.03	0.00	0.09	0.01	0.00

References

Amorndoljai, P. 2016. The use of ginger (Zingiber officinale Roscoe) in treatment and reloeves symptom of osteoarthritis. Journal of health and health management. 3(2), 13-22.

Anderson, E.F., 1993. Plants and People of the Golden Triangle Ethnobotany of the Hill Tribes of Northern Thailand. Timber Press, Inc., Southwest Portland

Charnprasert, C., Chansuwanit, N., 1992. Exploration and collection of medicinal plants in wasted areas of Muang district, Nonthaburi province. Kasetsart Journal (Natural Sciences) 26(1), 19.

Chaunchom, P., 2011. Ethnobotany of Hmong at Ban Tabboek, Tambon Wangban, Amphoe Lomkao, Changwat Phetchabun, Forest Resource Administration. Master thesis, Kasetsart University, Bangkok.

Chuakul, W., Saralamp, P., Boonpleng, A., 2002a. Medicinal plants used in the Kutchum district, Yasothon province, Thailand. Thai Journal of Phytopharmacy 9(1), 22-49.

Chuakul, W., Saralamp, P., Boonpleng, A., 2002b. Medicinal plants used in the Loengnoktha district, Yasothon province, Thailand. Thai Journal of Phytopharmacy 9, 23.

Chuakul, Wongsatit, and Ampol Boonpleng, 2003. Ethnomedical uses of Thai Zingiberaceous plant (1). Journal of Phytopharmacy. 10(1), 33-39.

Chuakul, W., Boonpleng, A., 2004. Survey on medicinal plants in Ubon Ratchathani province (Thailand). Thai Journal of Phytopharmacy 11, 33-54.

Chuakul, W., 2005. Medicinal plants in the Khok Pho district, Pattani province (Thailand). Thai Journal of Phytopharmacy 12(2), 23.

Chuakul, W., Soonthornchareonnon, N., Sappakun, S., 2006. Medicinal plants used in Kungkrabaen Royal Development Study Center, Chanthaburi province. Thai Journal of Phytopharmacy 13(1), 19.

Chuakul, W., 2012. Herbal plants for treatments of paralysis (*in Thai*). Thai Pharmaceutical and Health Science Journal. 5(3), 8.

Gunsuwan, P., 2011. Processes of transferring local wisdom on management of medicinal herbs diversity for traditional healing of Hmong Community : A Case study of Khun Chang Khian Village, Chang Phueak Sub-district, Mueang Chiang Mai District, Master thesis, Man and Environment Management, Chiang Mai University Library, Chiang Mai.

Imchan, T., 2006. Hmong's Wisdom in The Utilization And Conservation of Medicinal Plants in Bo Phak Subdistict Chattrakarn District Phitsanulok Province. Master thesis, Natural Resource and Environmental Management, Naresuan University Library, Phitsanulok.

Inta, A., 2008. Ethnobotany and crop diversity of Tai Lue and Akha communities in the upper northern Thailand and the Xishuangbanna Dai autonomous prefecture, China. PhD thesis, Biology, Chiang Mai University Library, Chiang Mai.

Inta, A., Panee, S.-A., Pongamornkul, W., 2013a. Medicinal plants in Ban Hua Thung community forest, Chiang Dao Wildlife Sanctuary, Chang Dao district, Chiang Mai province. Thai Journal of Botany 4(2), 20.

Inta, A., Trisonthi, P., Trisonthi, C. 2013b. Analysis of traditional knowledge in medicinal plants used by Yuan in Thailand. Journal of ethnopharmacology 149(1), 344-351. doi: https://doi.org/10.1016/j.jep.2013.06.047

Jearanai, J., 2012. A study of traditional thai complementary therapies, traditional Thai midwifery, and herbal utilization of traditional healers: a case study of traditional healers living in NumPung Dam, Sakon Nakhon province. Scientific report, Suranaree University of Technology Intellectual Repository, Nakhon Ratchasima.

Jitjum, S., Laohapraranon, S., Hayeeyahya, W., Khanittha, N. 2019. A study of traditional healers's herbal recipes for asthma treatment. J Med Health Sci. 26(3), 69-83.
Junkhonkaen, J., 2012. ethnobotany of Ban Bowee, Amphoe Suan Phueng, Changwat Ratchaburi. Master thesis, Kasetsart University, Kasetsart University Library, Bangkok.
Kantasrila, R. 2016. Ethnobotany of Karen in Ban Wa Do Gro, Mae Song subdistrict, Tha Song Yang district, Tak province. Master thesis. Chiagmai Univesity Library, Chaing Mai.
Kaewsangsai, S. 2017. Ethnobotany of Karen in Khun Tuen Noi Village, Mea Tuen subdistrict, Omkoi district, Chiang Mai province. Master thesis. Chiagmai Univesity Library, Chaing Mai.
Kaewwongsiri, P., Saiyapan, P. 2004. Medicinal plants in Khao Pra subdistrict, Rattaphum district, Songkla provice. Conservation and development of medicinal plant in Khao Pra project. Prince of Songkla University. Songkla.
Kesornmas, S., Nakthaworn, K., Musikapong, K., Viriyabubpa, C. 2019. A study on the treatment with herbal remedies in seven groups of diseases: A case study of Mr. Prawit Kaewthong, Songkhla Province. J Thai Trad Alt Med. 17(2), 263-279.
Khonkayan, S., Saengsiri, V., Thipsonthae, H. 2019. Medicinal plants in Phu Mae Nang Mon, Phu Sri Tan wildlife sanctuary, Mukdahan Province. Burapha Sci J. 24(2), 500-516.
Khuankaew, S., Srithi, K., Tiansawat, P., Jampeetong, A., Inta, A., Wangpakapattanawong, P., 2014. Ethnobotanical study of medicinal plants used by Tai Yai in Northern Thailand. Journal of Ethnopharmacology. 151(2), 829-838. doi: https://doi.org/10.1016/j.jep.2013.11.033
Maneenoon, K., Chatawatee, B., Damkhong, C., Khiankhan, N., Kunworarath, N. 2017. Knowledge o traditional healers on utilization of medicinal plants used for menstrual disorders in Krabi and Songkla provinces, Thailand. Burapha Sci J. 22(3), 243-258.
Muangyen, N., 2013. Ethnobotany of Tai Lue and Tai Yuan in Samoeng District, Chiang Mai Province. Master thesis, Biology. Chiang Mai University Library, Chiang Mai.
Neamsuvan, O., Jaisamut, P., Maneenoon, K., Subhateerasakul, S., 2012. A survey of medicinal plants for tonic from ban toong soong community forest, Auluk district, Krabi province. Burapha Science Journal 17(2), 7.
Neamsuvan, O., Boontong, J., Boonkaew, M., Sudrak, N., Moosigapong, K., 2013a. A study on indigenous knowledge of paresis-paralysis treatment from folk healers: A case study of Mrs. Somporn Sudjai, Chumphon Province. Thai Pharmaceutical and Health Science Journal 8(2), 8.
Neamsuvan, O., et al. 2013b. A Survey of Materia Medica for Rid-Si-Duang Healing: A Case Study of Rung Chumanee's Bud Khao Book, Nakhon Si Thammarat Province, Thailand. Thai Pharmaceutical and Health Science Journal 8(2): 8.
Neamsuvan, O., Sengnon, N., Haddee, U., Mard-E, W., Sae-Tang, W. 2014. Medicinal plants in tropical rain forest from Hua Khao subdistrict, Singha Nakhon district, Songkhla province, Thailand. AENSI. 8(5), 1-11.
Neamsuvan, O., Tuntien, S., 2015. Medicinal Plants used for women's healthcare from Khao Phanom Bencha National Park, Krabi Province. Burapha Science Journal 20(1), 15.
Neamsuvan, O., Komonhiran, P., Boonming, K. 2018. Medicinal plants used for hypertension treatment by folk healers in Songkhla province, Thailand. J Ethnopharmacol. 214(2018), 58-70. doi: https://doi.org/10.1016/j.jep.2017.11.032
Noitana, P., Saipara, S., Khoomput, K., 2013. Ethnobotany of the Hmong at Nanoi district, Nan province. Naresuan Phayao Journal 6(3), 7.
Nuammee, A., 2012. Ethnobotany of Hmong in Ban Pang Chang, Tambon Pong, Amphoe Santisuk, Changwat Nan. Master thesis, Botany. Chulalongkorn University, Bangkok.
Pannet, P., 2008. Life belief and ritual relating to herbal utilization : a medical ethobotanic study in Phu Thai ethnic group at Don Mai Khum village Kalasin province Thailand. Master thesis, Plant Science. Mahidol University, Bangkok.
Pantarod, B., 2002. A survey and collection of medicinal plants at Na Kwang village, Bo Kleau district, Nan province. Master thesis, Biology. Chiang Mai University Library, Chiang Mai.

Panyadee, P., Balslev, H., Wangpakapattanawong, P., Inta, A. 2019. Medicinal plants in homegardens of four ethnic groups in Thailand. J Ethnopharmacol. 239(2019), 11927. doi: https://doi.org/10.1016/j.jep.2019.111927

Panyaphu, K., 2012. Conservation and sustainable use of ethnomedicinal plants by Mien people in Nan Province. PhD thesis, Biology. Chiang Mai University Library, Chiang Mai.

Phueaknang, C., 2005. Ethnobotany of local people at Ban Chong Khaeb Samakkhee Forest, Tambon Tha Sao, Amphoe Sai Yok, Changwat Kanchanaburi. Master thesis, Forest Resource Administration. Kasetsart University, Bangkok.

Pipitkul, S., 2001. Medicinal plant utilization for the living of hill tribes at Doi Musoe, Tak Province. Master thesis, Technology of Environmental Management. Mahidol University Library and Knowledge Center, Bangkok.

Ponpim, Y., 1996. Ethnobotany of the hill trives in Kaenoy's and Nongkheuw's Royal Project in Chiang Mai. Master thesis, Biology. Chiang Mai University Libraly, Chiang Mai.

Poonthanaiwatkul, B., Lim, R.H.M., Howard, R.L., Pibanpaknitee, P., Williamsom, E.M. 2015. Traditional medicine use by cancer patients in Thailand. J Ethnopharmacol. 168(2015), 100-107.

Purintavaragul, C., Wongnawa, M., Thaina, P., 2012. Medicinal plants diversity in Kao-Pra Village, Thumbol Kao-Pra ,Amphoe Rattapoom, Songkhla Province. Thaksin Journal 15(2), 9.

Satean, G., Homhuan, S., Premprasit, S. 2018. Plant utilisation from Thungluilai community forest in Thungluilai subdistrict, Kornsan district, Chaiyaphum province. JSSNU. 14(2), 211-245. doi: 10.14456/jssnu.2018.19

Sinworn, S., Viriyawattana, N., 2014. The diversity of medicinal plants and utilization in Khoa Phra, Doembangnangbuat District, Suphanburi Province. SDU Research Journal Sciences and Technology 7(1), 19.

Siriyong, T., Phungtammasan, S., Jansorn, S., Chonsongkram, N., Chanwanitsakul, S., Subhadhirasakul, S, Voravuthikunchai, S.P. 2019. Traditional Thai herbal medicine as an alternative treatment for refractory chronic eczema. Explore. 000(2019), 1-8. doi: https://doi.org/10.1016/j.explore.2019.10.001

Sonsupub, B., 2010. Ethnobotany of karen community in Raipa village, Huaykhayeng subdistrict, Thongphahpume district, Kanchanaburi province. Master thesis, Hoticulture. Kasetsart University, Bangkok.

Srisanga, P., Wongpakam, S., Kamkuan, W., Pekthong, T., Tovaranonte, J., Yaso, T., Nontachaiyapoom, S., 2011. Ethnobotany of Akha in Huay Yuak Pa So village, Mae Fah Luang district and Ban Mai Patthana village, Mae Suai district, Chiang Rai province. Thai Journal of Botany 3(1), 12.

Srithi, K., 2012. Comparative ethnobotany in Nan province, Thailand. PhD thesis, Biology. Chiang Mai University Library, Chiang Mai.

Sukkho, T., 2008. A survey of medicinal plants used by Karen people at Ban Chan and Chaem Luang Subdidtricts, Mae Chaem district, Chiang Mai province. Biology. Chiang Mai University Library, Chiang Mai.

Suwanantawong, N., 2011. Pharmaceutical ethnobotanical study of Thai-Khmer ethnic group in 2 villages in the Khukhan district, Si Sa Ket province. Master thesis, Plant

Tangtragoon, T., 1998. Ethnobotany of the Khamu, Lawa and H'tin in some areas of Nan Province. Master thesis, Biology. Chiang Mai University Library, Chiang Mai.

Thaenkham, A., Tiyaworanant, S., Padumanoda, T. 2019. The ethnobotanical survey of traditional plant of Pamanow subdistrict, Banfang district, Khon Kaen province. Udon Thani Rajabhat University J Sci Tech. 7(2), 61-75.

Thangthaisong, T., Caichompoo, W., Phadungkit, M., 2011. The ethnobotanical survey of plants used for treatment of bone disorders in Amphur Muang, Mahasarakram Province. Thai Pharmaceutical and Health Science Journal 6(1), 5.

Tichachart, C. 2004. Ethnobotany of Hmong hilltribe in Tambon Kheknoi, Amphur Khaokor, Changwat Phetchabun. Master thesis. Kasetsart University, Bangkok.

Tovaranonte, J., 1998. Ethnobotanical study of the Tai Lue, Hmong and Yao in Some areas of Nan Province. Master thesis, Biology. Chiang Mai University Library, Chiang Mai.

Upho, U., 2005. Ethnobotany of buddhist and muslim Thais in some locations in the lower part of southern Thailand. PhD thesis, Biology. Chiang Mai University Library, Chiang Mai.

Wannasri, S., Buapan, P. 2013. Diversity and utilization of medicinal plants in the Pho-Phang-Mar Lom Khao district Phetchabun province. NRU & HERB. Office of the higher ecocation commission. Ministry of Education. Bangkok.

Wayo, Y., 2012. A study on herbal plants used for healing of Ban Wang Phai community, Wichian Buri district, Phetchabun province. Master thesis, Education of Environment. Uttaradit Rajabhat University, Uttaradit.

Winijchaiyanan, P., 1995. Ethnobotany of Karen in Chiang Mai. Master thesis, Biology. Chiang Mai University Library, Chiang Mai.

Yaso, T., 2000. Ethnobotany of the H'tin and Lua in Phuphaa subdistrict, Baugleua district, Nan Province. Master thesis, Biology. Chiang Mai Univesity Library, Chiang Mai.
